# Exercise snacks in university settings: a mini review of daily physical activity and sedentary behavior among university students

**DOI:** 10.3389/fpsyg.2026.1928216

**Published:** 2026-10-26

**Authors:** XiaoTian Wang, JingLin Wu, Liang Heng, HaoRan Li, ZeYu Zou

**Affiliations:** 1 College of Education for the Future, Beijing Normal University, Zhuhai, China; 2 Faculty of Health Sciences and Sports, Macao Polytechnic University, Macao, China; 3 College of Physical Education and Sports, Beijing Normal University, Beijing, China; 4 School of Physical Education, Guangdong University of Education, Guangzhou, China; 5 Physical Education Center, Nanfang College·Guangzhou, Guangzhou, China

**Keywords:** activity breaks, exercise snacks, physical activity, sedentary behavior, university health promotion, university students

## Abstract

University students spend much of their daily life in classrooms, online learning, screen use, library study, dormitory routines, and campus commuting, making insufficient physical activity and prolonged sitting important concerns for health promotion in higher education. Exercise snacks, defined here as brief, repeated, low-barrier bouts of physical activity that can be embedded in ordinary routines, have attracted growing attention in exercise health, public health, and sedentary behavior research. Compared with physical education courses or structured exercise, exercise snacks use short gaps in learning and daily life, for example through brisk walking, stair climbing, dynamic stretching, body-weight exercise, or brief moderate-to-vigorous activity, to increase movement opportunities and interrupt prolonged sitting. This mini review adopts an integrative thematic approach to synthesize work on exercise snacks, physical activity snacks, brief intermittent physical activity, active breaks, sedentary behavior interruption, incidental physical activity, Brain Breaks, and physical activity among university students. It discusses conceptual boundaries, potential effects, contextual conditions, and future directions for translating exercise snacks into university settings. The literature suggests that exercise snacks should be viewed as a supplementary strategy alongside physical education and structured exercise. They may help increase daily physical activity, reduce or interrupt prolonged sitting, and improve the distribution of activity across the day. However, the field is marked by inconsistent definitions, a limited body of university-specific research, scarce evidence from real campus contexts, and insufficient objective measurement. Future studies should define exercise snacks operationally for higher education, prioritize safe, feasible low- to moderate-intensity activities, test interventions within real timetables and campus spaces, and combine wearable devices, smartphone step counts, and activity logs to assess physical activity and sedentary behavior. Exercise snacks in university settings should therefore not be framed as a replacement for physical education, but as a low-cost, accessible, context-embedded pathway for promoting daily physical activity.

## 1 Introduction

University students’ everyday lives have become increasingly sedentary. Classroom learning, online courses, screen use, library study, dormitory-based leisure, and campus commuting together shape students’ daily activity patterns. Prolonged sitting is associated with disease incidence, mortality risk, and adverse health outcomes, and extended sedentary time may remain harmful even among people who achieve some physical activity (Biswas et al., 2015; Ekelund et al., 2019). Among university students, insufficient physical activity and sedentary behavior are also shaped by gender, age, field of study, degree level, academic arrangements, and campus environments (Castro et al., 2018, 2020; Edelmann et al., 2022; Vella and Nelson, 2024; Yuan et al., 2024). Castro et al. reported markedly higher sitting time when measured by accelerometry than by self-report, suggesting subjective underestimation. Physical activity and sedentary behavior are also linked to academic performance, mental health, and wellbeing, indicating that activity promotion in higher education belongs within campus health promotion and youth public health interventions (Babaeer et al., 2022; Barbosa et al., 2024).

University physical activity promotion usually relies on physical education courses, extracurricular sport, sport clubs, campus competitions, and health education. These approaches are valuable for fitness, movement skills, and campus sport culture, but they require time, space, and organization. Digital health and campus-based interventions can improve activity among college students, although effects vary by format, duration, measures, and maintenance (Peng et al., 2022). Limited physical education classes or long extracurricular sessions alone are therefore unlikely to address the small, dispersed movement gaps in students’ daily routines.

Exercise snacks offer a different approach. Early work showed that short activity bouts may benefit metabolic health; for example, pre-meal exercise snacks improved glycemic control in individuals with insulin resistance (François et al., 2014). Islam et al. defined exercise snacks as isolated, periodic vigorous bouts lasting no longer than 1 min across the day, while Jones et al. described exercise snacks and related intermittent activities as emerging activity-promotion strategies with varied definitions, duration, intensity, frequency, and outcomes (Islam et al., 2022; Jones et al., 2024). Snacktivity research similarly suggests that embedding 2–5 min activity bouts into daily life may offer an acceptable and accumulable strategy for insufficiently active populations (Sanders et al., 2021; Daley et al., 2023). The value of exercise snacks is therefore not merely shorter exercise time, but the reorganization of activity into small, repeatable, context-fit opportunities.

For university students, exercise snacks can occur between classes, during building transitions, in library study, in dormitory life, during campus commuting, or after sustained seated study. Brisk walking, stair climbing, dynamic stretching, and low-intensity body-weight exercises can increase movement opportunities and break up sitting. Stair-climbing exercise snack studies suggest that brief, dispersed stair climbing may improve cardiorespiratory fitness in inactive young adults, supporting the possible use of low-cost campus spaces such as stairways (Jenkins et al., 2019; Yin et al., 2024). Yet evidence based on students’ real campus lives remains limited (Weston et al., 2025).

This review does not treat exercise snacks as a replacement for physical education or structured exercise. It understands them as a supplementary strategy embedded in ordinary university routines and examines their conceptual boundaries, potential effects, contextual conditions, and future research directions.

## 2 Review scope and literature basis

This mini review uses an integrative thematic approach to organize the literature on exercise snacks in university settings and their potential value for promoting daily physical activity. It does not pool effect sizes, conduct formal quality appraisal, or claim an overall causal effect. The literature instead supports four lines of discussion: definitions of exercise snacks and adjacent concepts; evidence on brief activity and sedentary behavior interruption; insufficient activity and sedentary behavior among university students; and the feasibility and measurement of micro-activity interventions in real campus settings.

The literature base was drawn mainly from English-language peer-reviewed studies retrievable in PubMed, Web of Science, and Scopus. Recent studies published between 2020 and 2025 were prioritized to capture current work on exercise snacks, Snacktivity, university-student sedentary behavior, classroom activity breaks, and wearable-based measurement. Representative studies from 2012 to 2019 were retained when foundational for sedentary behavior interruption, the origins of exercise snacks, brief activity and health, or digital activity measurement. Search terms included exercise snacks, physical activity snacks, movement snacks, Snacktivity, brief intermittent physical activity, incidental physical activity, brain breaks, sedentary behavior interruption, active breaks, movement breaks, breaking up prolonged sitting, university students, college students, higher education, daily physical activity, sedentary behavior, wearable devices, and accelerometry.

Because this research remains at an early stage, the literature does not yet allow firm conclusions about effectiveness. The review therefore integrates studies on exercise snacks, physical activity snacks, brief activity bouts, incidental physical activity, movement-based Brain Breaks, classroom activity breaks, sedentary behavior interruption, vigorous intermittent lifestyle physical activity (VILPA), university-student physical activity interventions, and wearable-based measurement.

## 3 Conceptual boundaries, potential effects, and contextual conditions

Brief, dispersed, and accumulable activity bouts are becoming important in physical activity promotion. Exercise snacks, physical activity snacks, brief intermittent physical activity, active breaks, and sedentary behavior interruption originate from different research lines, but they share the premise that activity can be embedded in everyday life in small units rather than relying only on facilities, uninterrupted time, or structured training. Reviews and meta-analyses suggest favorable effects on some adult cardiorespiratory and cardiometabolic indicators, while designs, doses, and outcomes remain heterogeneous (Jones et al., 2024; Chen et al., 2025). Snacktivity studies also suggest that 2–5 min activity snacks have public health value and show feasibility and acceptability (Sanders et al., 2021; Daley et al., 2023, 2025; Krouwel et al., 2023).

### 3.1 Conceptual boundaries: meaning and scope of exercise snacks in higher education

In university settings, exercise snacks can be understood as brief, repeatable, low-equipment bouts of physical activity undertaken between classes, during study breaks, in campus commuting, during building transitions, or in dormitory life. They differ from physical education courses and structured exercise, which are oriented toward systematic training or sport-specific skill learning. They also differ from traditional class-break exercises that depend on unified organization and standardized movements. Compared with ordinary walking or commuting, exercise snacks emphasize the intentional use of short time gaps to increase activity stimuli and reduce continuous sitting. The term therefore does not refer to every brief movement on campus, but to short activity bouts that have a clear activity intention, can be repeated, and are connected to specific campus contexts.

Related concepts include incidental physical activity (IPA) and Brain Breaks. IPA occurs incidentally during daily activities such as commuting, building transitions, or stair use, without exercise as the primary purpose (Stamatakis et al., 2019). Movement-based Brain Breaks use brief standing, stretching, or simple movements to interrupt sedentary study (Mok et al., 2020; Lynch et al., 2022). Although overlapping with exercise snacks, they are not equivalent: exercise snacks emphasize intentional, repeated brief activity, whereas IPA and movement-based Brain Breaks provide complementary models for daily-life and learning contexts.

Given differences in students’ physical condition and campus constraints, the duration of each bout can provisionally be set at 1–5 min, with intensity ranging from light to moderate or higher intensity. Light forms may include standing activity, dynamic stretching, and easy walking; moderate forms may include brisk walking, stair climbing, and body-weight exercises; higher-intensity forms may include short bouts of jumping jacks, fast step-ups, squat jumps, or combined interval movements. This layered understanding avoids reducing exercise snacks to high-intensity interval training, while also preventing the overextension of the concept to any daily movement. Research on VILPA shows that brief episodes of higher-intensity activity in daily life can be identified and measured, and may carry health significance, which provides adjacent evidence for understanding short activity bouts in campus life (Stamatakis et al., 2022).

Exercise snacks in higher education should not be equated directly with the prototype definition proposed by Islam et al., namely high-intensity exercise bouts lasting no longer than 1 min (Islam et al., 2022). They are better understood as a contextual translation of that concept into students’ daily learning and living routines. University health promotion needs to consider not only the physiological stimulus of brief activity, but also differences in student fitness, class-break duration, spatial safety, acceptability, and long-term executability. Exercise snacks in higher education are therefore best defined as a practically flexible strategy of brief physical activity. They stand in a supplementary, rather than substitutive, relationship to physical education courses, extracurricular exercise, and systematic training.

### 3.2 Potential effects: daily physical activity, sitting interruptions, and activity distribution

Within these boundaries, the potential effects of exercise snacks can be understood primarily at the behavioral level. Rather than relying on one bout to produce a strong training effect, accumulated brief bouts may influence total activity, sitting-break frequency, and the distribution of activity across the day. Because university-based evidence remains nascent, the analysis draws on studies of exercise snacks, sedentary behavior interruption, classroom activity breaks, and university-student physical activity.

University campuses are spatially mobile environments. Students regularly move among dormitories, teaching buildings, libraries, dining halls, and sport facilities. Inserting intentional brisk walking, stair climbing, body-weight exercise, or dynamic stretching into existing routes may increase steps, light-to-moderate activity, and activity frequency. Compared with adding a complete exercise session, this approach uses existing routines and better fits fragmented timetables. E-health and campus-based interventions can improve steps, total physical activity, or moderate-to-vigorous physical activity, although effects depend on format, duration, and implementation context (Peng et al., 2022).

A second potential value is interrupting prolonged sitting. Students often sit for long periods in classes, libraries, and screen-based dormitory activities. Risk is related not only to total sitting time but also to its distribution. Brief activity can shift the body temporarily out of a static state and increase sitting breaks through standing, walking, stretching, brisk walking, or stair climbing. Research suggests that more frequent breaks are associated with some cardiometabolic indicators, and that standing, light walking, or simple resistance activity may improve postprandial glucose, insulin responses, and some metabolic markers. Effects vary by break type and dose (Dunstan et al., 2012; Chastin et al., 2015; Dempsey et al., 2016; Buffey et al., 2022).

Exercise snacks may also shift activity from concentrated sessions to a distributed daily rhythm. The World Health Organization 2020 guidelines no longer require adult physical activity to be accumulated in bouts of at least 10 min, bringing short bouts into health promotion (Bull et al., 2020). Evidence on bout duration and health similarly indicates that benefits need not depend on longer continuous bouts (Jakicic et al., 2019). Exercise snacks may therefore help distribute movement across multiple learning and living nodes, although whether repeated occurrence in stable campus contexts strengthens behavioral consistency requires longitudinal verification.

These potential effects require caution. Existing exercise snack studies involve mainly adult, older adult, or general sedentary populations, and vary in intensity, duration, frequency, and intervention period. Operational definitions, activity forms, health outcomes, study quality, samples, and intervention designs remain inconsistent (Jones et al., 2024; Chen et al., 2025). Exercise snacks in higher education are therefore a promising campus-based strategy rather than a validated standardized prescription. Their effects on steps, moderate-to-vigorous activity, sitting interruptions, and adherence require testing in real universities.

### 3.3 Contextual conditions: timetables, campus spaces, and digital measurement

Universities provide several contextual conditions for exercise snack interventions. Timetables contain short windows between classes, after consecutive theoretical classes, and during long study sessions. Campus spaces such as stairways, corridors, sport-field edges, dormitory roads, library surroundings, and greenways can accommodate brisk walking, stair climbing, dynamic stretching, or body-weight exercises. Activity-break research in higher education shows that standing or classroom movement breaks are feasible in university courses and may reduce sedentary time, relieve fatigue, or improve perceived focus (Paulus et al., 2021; Peiris et al., 2021; Lynch et al., 2022).

Digital tools make brief activity bouts easier to record. Because exercise snacks are short, questionnaires may capture their frequency and cumulative effects poorly. Future studies should combine wearable devices, smartphone step counts, and activity logs to assess steps, moderate-to-vigorous activity, sitting interruptions, and activity rhythms. Brief higher-intensity daily activity can be device-detected, and wearables and smartphone applications may improve monitoring and increase steps and moderate-to-vigorous activity, although effects on sedentary behavior and maintenance need further confirmation (Gal et al., 2018; Stamatakis et al., 2022; Longhini et al., 2024).

Universities are not homogeneous. Class-break length, building structure, stairway safety, curriculum density, student fitness, and campus management cultures differ across institutions. Some spaces are unsuitable for higher-intensity activity, and some students may need breaks for rest, social interaction, or preparation. Overly compulsory designs or uniform check-in tasks may reduce acceptability. Qualitative Snacktivity work suggests that acceptability depends on individual perception, contextual fit, activity choice, and flexibility (Krouwel et al., 2023). Future designs should therefore emphasize voluntariness, flexibility, safety, and moderation.

## 4 Research gaps and future directions

Research on exercise snacks, Snacktivity, sedentary behavior interruption, VILPA, and wearable-based measurement offers important reference points, but exercise snacks in higher education remain at the stage of conceptual translation and contextual validation. The urgent task is to clarify what counts as an exercise snack in university settings and whether short bouts can alter the distribution of physical activity and sedentary behavior in real university life (Jones et al., 2024; Weston et al., 2025).

Terms such as exercise snacks, physical activity snacks, movement snacks, active breaks, and sedentary behavior interruption overlap, but differ in duration, intensity, setting, and target outcome. For university students, exercise snacks should neither be equated with high-intensity bouts lasting no longer than 1 min nor generalized to any campus walking. Future studies should specify duration, intensity, frequency, and contextual boundaries in relation to timetables, spaces, and daily rhythms, making protocols more repeatable and comparable.

Direct evidence from real universities remains limited. Much evidence comes from adults, office workers, older adults, metabolic-risk populations, or general sedentary groups, whereas university students have distinctive study rhythms, spatial mobility, and residential arrangements. Student studies suggest that activity and sedentary patterns are influenced by gender, age, field of study, degree stage, and everyday context, and that interventions can improve total activity, moderate-to-vigorous activity, and steps (Edelmann et al., 2022; Peng et al., 2022; Vella and Nelson, 2024; Yuan et al., 2024). These findings do not directly establish exercise snack effects in campus settings. Future research should prioritize device-measured activity, sitting interruptions, and prolonged sitting, while examining acceptability, safety, and maintenance across class breaks, teaching buildings, libraries, dormitories, and campus commuting. Classroom activity-break studies provide adjacent evidence, but the scope should extend to fuller daily-life patterns (Paulus et al., 2021; Peiris et al., 2021; Lynch et al., 2022).

Implementation should consider intensity, context, and individual differences. For most students, simple, equipment-free low- to moderate-intensity activities (e.g., brisk walking, stretching, stair climbing, or body-weight exercise) can be adapted to class breaks, study, dormitory life, or commuting. Movement-based Brain Breaks may be incorporated during prolonged study, provided they do not disrupt learning, while IPA-informed strategies can make use of routine commuting and building transitions (Stamatakis et al., 2019; Lynch et al., 2022). Higher-intensity activity should not be uniformly recommended; fitness, health status, preparation, and site safety must be considered. Future studies should compare intensities, frequencies, and contexts for feasibility, acceptability, and effectiveness, informing safe, flexible, sustainable protocols.

## 5 Discussion

This mini review synthesized the potential value of exercise snacks for promoting daily physical activity among university students. Exercise snacks should not be understood as a replacement for physical education or structured exercise, but as a supplementary strategy embedded in ordinary learning and living routines. Their value lies not in the training effect of any single bout, but in repeated, contextualized opportunities for movement and interruption of prolonged sitting.

This review extends exercise snacks from adult health promotion and cardiometabolic research into daily activity promotion among university students. While existing work has focused largely on cardiorespiratory fitness, metabolic health, or physical function, the higher education setting foregrounds how brief activity can enter timetables, campus spaces, and study breaks. The central argument is that exercise snacks are not merely miniaturized exercise, but a contextualized form of activity promotion. They represent a shift from an exercise-prescription logic to a lifestyle-embedding logic: by reorganizing the time gaps and spatial opportunities of university life, they may make activity easier to incorporate into daily routines. This position aligns with public health emphasis on reducing prolonged sitting, increasing activity breaks, accumulating activity across the day, and using digital devices to monitor real-life behavior (Chastin et al., 2015; Bull et al., 2020; Stamatakis et al., 2022; Longhini et al., 2024).

This review has limitations. Its integrative thematic approach aimed to clarify concepts, contexts, and directions rather than systematically evaluate intervention effects. It did not conduct a strict systematic search, quality appraisal, or effect-size synthesis. Direct empirical research in higher education remains limited, and some inferences are based on thematic links among exercise snacks, Snacktivity, sedentary behavior interruption, classroom activity breaks, university-student physical activity, and wearable-based measurement. Studies also vary in intensity, duration, frequency, measurement, and outcomes; thus, the optimal dose and actual effectiveness remain undetermined.

Future research should test feasibility, safety, and differential effects in real campuses before exercise snacks can become a replicable activity-promotion strategy. More broadly, their frontier value is not a shorter training model, but a prompt to reconsider the basic unit and setting of physical activity promotion. Activity need not always depend on complete courses, fixed venues, or long sessions; it can enter university life through brief, dispersed, and repeatable forms. This review therefore does not argue for replacing university physical education. It suggests that health promotion should attend both to completing exercise sessions and to distributing physical activity across the day, shifting activity toward campus-life nodes that can be identified, measured, and repeated.
